# Accumulation of Circulating Cell-Free CpG-Enriched Ribosomal DNA Fragments on the Background of High Endonuclease Activity of Blood Plasma in Schizophrenic Patients

**DOI:** 10.1155/2019/8390585

**Published:** 2019-08-05

**Authors:** E. S. Ershova, E. M. Jestkova, A. V. Martynov, G. V. Shmarina, P. E. Umriukhin, L. V. Bravve, N. V. Zakharova, G. P. Kostyuk, D. V. Saveliev, M. D. Orlova, M. Bogush, S. I. Kutsev, N. N. Veiko, S. V. Kostyuk

**Affiliations:** ^1^Research Centre for Medical Genetics (RCMG), Moscow 115478, Russia; ^2^I.M. Sechenov First Moscow State Medical University (Sechenov University), Moscow, Russia; ^3^Psychiatric Hospital No. 4 of Moscow City Health Department, Moscow 115447, Russia; ^4^P.K. Anokhin Institute of Normal Physiology, Moscow, Russia; ^5^N.A. Alexeev Clinical Psychiatric Hospital No. 1 of Moscow Healthcare Department, Moscow 115447, Russia; ^6^Rowan University Biological Sciences Department, Science Hall, Glassboro, New Jersey, USA

## Abstract

**Introduction:**

Schizophrenia (SZ) increases the level of cell death, leading to an increase in the concentration of circulating cell-free DNA (cfDNA). Ribosomal DNA (rDNA) contains many unmethylated CpG motifs that stimulate TLR9-MyD88-NF-*κ*B signaling and the synthesis of proinflammatory cytokines. The number of rDNA copies in the genomes of SZ patients is increased; therefore, we expect that the concentration of cell-free rDNA in the plasma of the SZ patients also increases. This may be one of the explanations of the proinflammatory cytokine increase that is often observed in SZ. The major research question is what is the rDNA copy number in cfDNA (cf-rDNA CN) and its putative role in schizophrenia? *Materials and Methods*. We determined cfDNA concentration (RNase A/proteinase K/solvent extraction; fluorescent dye PicoGreen) and endonuclease activity (NA) of blood plasma (radial diffusion method) in the untreated male SZ group (*N* = 100) and in the male healthy control group (HC) (*N* = 96). Blood leukocyte DNA and cfDNA rDNA CN were determined with nonradioactive quantitative hybridization techniques. Plasma concentration of cf-rDNA was calculated.

**Results:**

In the subjects from the SZ group, the mean cfDNA plasma concentration was twofold higher and NA of the plasma was fourfold higher than those in the healthy controls. rDNA CN in the blood leukocyte genome and in the cfDNA samples in the SZ group was significantly higher than that in the HC group. cf-rDNA concentration was threefold higher in the SZ group.

**Conclusion:**

Despite the abnormally high endonuclease activity in the blood plasma of SZ patients, the circulating cfDNA concentration is increased. Fragments of cf-rDNA accumulate in the blood plasma of SZ patients. Potentially, SZ patients' cfDNA should be a strong stimulating factor for the TLR9-MyD88-NF-*κ*B signaling pathway.

## 1. Introduction

Schizophrenia (SZ) is a neurodevelopmental disorder that is associated with deficits in cognition, affect, and social functioning. The SZ morbidity in the population differs slightly in the different world regions and reaches 1.45% [1]. The morbidity is largely influenced by changes in the genome, but the onset of the disease significantly depends on environmental factors and the presence of stress in the patient's life. Heritability of schizophrenia is about 80%. It is believed that many genes are involved in the development of the disease, changes in each of them may have a small effect on the pathogenesis, and they act in conjunction with epigenetic disorders and environmental factors [2]. Despite the high heritability, a large proportion of people with schizophrenia do not have a family history of the disease (sporadic cases) [3].

Recently, we discovered that genomes of some SZ patients contain more ribosomal genes than the healthy controls [4]. The diploid human genome contains ∼400 copies of a 43 kb rDNA unit tandemly arrayed in nucleolar organizer regions on the five acrocentric chromosomes. Each unit contains 13.3 kb of a sequence encoding the 28S, 5.8S, and 18S rRNAs (45S rRNA) and a noncoding intergenic spacer (IGS) [5]. Together with the 5S rRNA (encoded by genes located on chromosome 1), these rRNAs form the nucleic acid backbone of the ribosome. Possibly, an increased number of ribosomal gene copies in the human genome may be one of the factors for the schizophrenia development [4].

Abnormal redox homeostasis, oxidative stress, and neuroinflammation have been proposed to play a role in the SZ etiology [6]. It is suggested that oxidative stress plays a role in schizophrenia through oxidative DNA damage [7]. Cells with damaged DNA die and may be one of the main sources of circulating cell-free DNA (cfDNA). It is shown that exposure to chronic psychosocial stress influences plasma cfDNA levels [8–10]. In some recent studies, elevated cfDNA levels were found in schizophrenia patients that evidently reflect increased apoptotic activity observed during the disease [11, 12]. Authors suppose that in the future, cfDNA may serve as an auxiliary diagnostic marker.

It is important to note that cfDNA should be regarded as “a danger-associated molecular pattern” molecule (DAMP) [13, 14]. The range of DAMPs is quite wide. In the healthy cell, potential DAMPs can perform various physiologic functions. However, upon their release from cells during injury, these endogenous molecules can acquire immunologic activity and, instead of their ordinary function, become danger signals. Mammalian self-DNA was for a long time deemed to exert no influence on the immune system cells. However, this point of view changed in recent years. Significant difference was found between cfDNA and genomic DNA (gDNA) in the content of certain motifs [15–17], degree of oxidative modification of the bases [12], and methylation level [18]. It has been shown that GC-rich motifs are accumulated in the total pool of cfDNA with time [19, 20]. The average GC pair content is 53.7% in the cfDNA [20], whereas the GC pair content is 42% in the nuclear DNA ([21]). GC-rich motifs can stimulate TLR9 receptors. The TLR9 receptor can recognize unmethylated CpG DNA motifs [22]. The TLR9 immune signaling induces expression of genes mediated by nuclear factor kappa B (NF-*κ*B) [23, 24].

The transcribed ribosomal repeat region (TR-rDNA) is one of the most GC-rich genome sequences. The nontranscribed spacer is not characterized by the high GC pair content, while various areas of human TR-rDNA contain from 60% to 85% of the GC pairs. Cell-free TR-rDNA is a potential ligand for TLR9 [16]. The TR-rDNA content is considerably increased in cases of some chronic diseases and conditions, such as coronary heart disease [25], rheumatoid arthritis [16], and chronic external damaging impact (for example, a heightened ionizing radiation [26]). In these cases, as a rule, the total cfDNA level in blood plasma of the affected subjects becomes unchanged or lowered, compared to the normal baseline, due to a noticeable increase in the endonuclease activity of the blood plasma [25, 27, 28]. We have shown previously that the increase of the TR-rDNA concentration in the culture medium of healthy human lymphocytes leads to the stimulation of TLR9-MyD88-NF-*κ*B signaling and to the synthesis of significant amounts of proinflammatory cytokines IL-6 and TNFa [29]. However, a common inflammatory process involving cytokine imbalance is associated with symptoms of schizophrenia; high concentrations of IL-6 are tested in the plasma of patients. A higher concentration of IL-6 correlated positively with greater cognitive impairments [30].

Possibly, the increased rDNA CN in the genomes of SZ patients [4] leads to an increase in the cell-free TR-rDNA concentration in the blood plasma of patients compared with healthy controls, which in turn can affect the synthesis of proinflammatory cytokines. In the present study, we determined the cfDNA concentration, blood plasma endonuclease activity, and the content of the TR-rDNA fragment in cfDNA and in the blood plasma of the untreated SZ patients and healthy controls.

## 2. Materials and Methods

### 2.1. Subjects

The investigation was carried out in accordance with the latest version of the Declaration of Helsinki and approved by the Regional Ethics Committee of RCMG (approval #5). All participants signed an informed written consent to participate after the nature of the procedures had been fully explained to them. 100 male paranoid SZ patients with acute psychotic disorders were recruited from general psychiatric units for the treatment of acute forms of mental disorders (Psychiatric Hospitals #1 and #14 of Moscow City Health Department, Moscow). Demographic and clinical measures in the male SZ patients and male HC are presented in Table 1. Psychopathology and functionality of patients were measured according to the Positive and Negative Syndrome Scale (PANSS). Patients were diagnosed with paranoid schizophrenia (F20.00 or F20.01) according to ICD-10 criteria using structured interviews (MINI). Diagnoses were also confirmed pursuant to DSM-IV criteria. 45 patients did not take the medication for at least two weeks before the venous blood was sampled. 55 patients were diagnosed as the first-episode SZ and were never treated with antipsychotic medications. No subject had comorbidities. The control group consisted of 96 healthy males with no history of any psychiatric disorder and age matched to the patient group. There were no statistical differences between the examined groups in terms of smokers' frequencies.

### 2.2. DNA Isolation from the Blood

Five mL of the blood was collected from the peripheral vein with a syringe flushed with heparin (0.1 mL/5 mL blood) under strict aseptic conditions. Cells were separated from the blood by centrifugation at 460 × g. Cellular debris was removed by centrifugation at 16.000 g for 10 min. DNA was isolated from leukocytes (g-DNA) and plasma (cfDNA) by the standard method [4, 27]. Briefly, 3 mL of plasma or cell suspension was mixed with 0.3 mL of the solution containing 1% sodium lauryl sarcosylate, 0.02 M EDTA, and 75 *μ*g/mL RNAse A (Sigma, USA), incubated for 45 min, then treated with proteinase K (200 *μ*g/mL, Promega, USA) for 24 h at 37°C. After two cycles of the purification with saturated phenolic solution, DNA fragments were precipitated by adding two volumes of ethanol in the presence of 2 M ammonium acetate. The precipitate was then washed with 75% ethanol twice and dissolved in water. The DNA quantification (index cfDNA) is determined fluorimetrically using the PicoGreen dsDNA quantification reagent by Molecular Probes (Invitrogen, CA, USA). The assay displays a linear correlation between dsDNA quantity and fluorescence within a wide range (1-100 ng/*μ*L) of concentrations (Supplement (available here)). The DNA concentration in the sample is calculated according to a DNA standard curve. We use Enspire equipment (Finland) at excitation and emission wavelengths of 488 and 528 nm, respectively. The relative standard error of the index cfDNA was 10 ± 4%.

### 2.3. Analysis of Blood Plasma Endonuclease Activity

We applied a standard radial diffusion method, described in detail previously [31]. To calculate the endonuclease activity, a calibration dependence was obtained, which relates the fluorescence of the dye in the spot with the concentration of the standard DNase 1 sample (Sigma) in solution. The result is given in units of activity (U/mL). 1 unit corresponds to the activity of DNase1, taken at a concentration of 1 ng/mL (1 hour, 37°C). At least three parallel measurements were made for one sample. The relative standard error of the method is 5%.

### 2.4. Nonradioactive Quantitative Hybridization (NQH)

The method of quantitative nonradioactive hybridization was specified in detail previously [4, 26]. The method details are shown in the Supplement. The relative standard error for NQH only was 5 ± 2%. The main contribution to the overall error of the experiment is made by the step of isolating DNA from the leukocytes. The total standard error was 11 ± 8%.

### 2.5. Statistical Analysis

All the findings reported here were reproduced at least two times as independent biological replicate. The descriptive statistics are listed in Table 1. The significance of the observed differences was analyzed using the nonparametric Mann-Whitney *U* test and the Kolmogorov-Smirnov statistics. Linear regression analyses were carried out to evaluate the effect of cfDNA as a function of NA. Data were analyzed with StatPlus 2007 Professional software (http://www.analystsoft.com/). All *p* values were two sided and considered statistically significant at *p* < 0.05.

## 3. Results

One hundred male SZ patients not taking antipsychotic drugs at the time of blood tests were included in the study (SZ group). Table 1 lists demographic and clinical measures in the SZ patients. Ninety-six healthy male volunteers who had no psychotic episodes in the past were used for control (HC group). Five parameters reflecting the properties of plasma cell-free DNA (cfDNA) were determined: the concentration of cfDNA and cf-rDNA (index cfDNA and index cf-rDNA), the content of ribosomal repeat in the DNA of blood leukocytes and plasma cfDNA (index g-rDNA CN and index cf-rDNA CN), and index *R* = cf-rDNA CN/g-rDNA CN. In addition, endonuclease activity of blood plasma (index NA) as a factor of the cfDNA elimination from the bloodstream was determined.

### 3.1. cfDNA and NA of the Blood Plasma Increase in the SZ Patients

**Figure 1:**
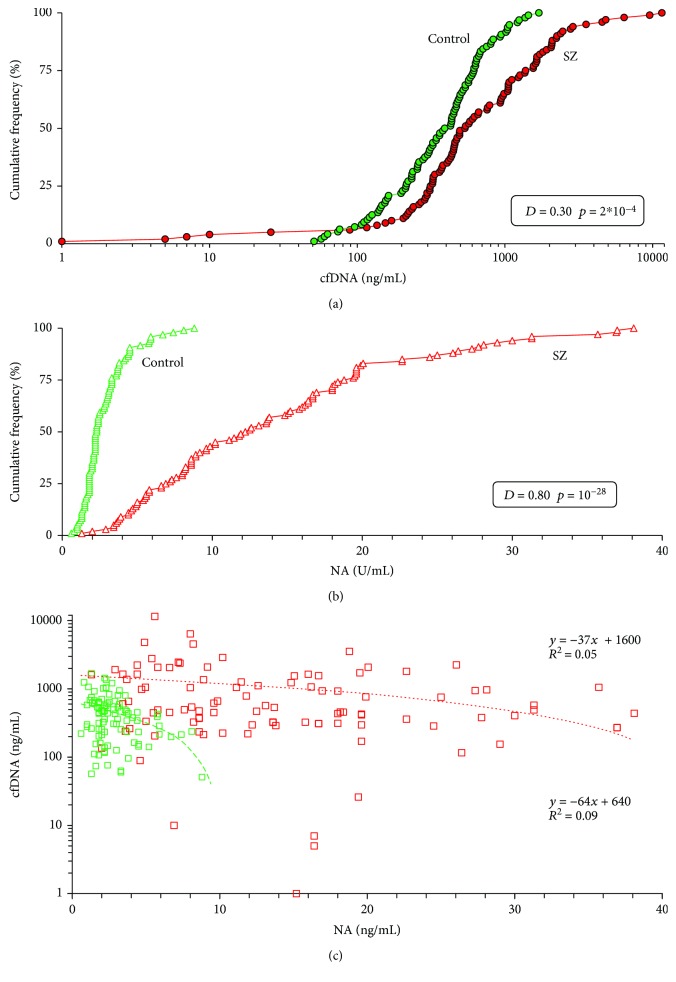
In the untreated male SZ patients, plasma cfDNA concentration and endonuclease activity are increased. (a) Cumulative curves (distributions) of the cfDNA concentrations in the control group (*N* = 96) and the SZ group (*N* = 100). Table presents the data of the Kolmogorov-Smirnov statistics nonparametric for pairwise comparison of the two groups.  Table 2 (line 1) presents the descriptive statistics and comparison of two samples using the *U* test. (b) Cumulative curves of the index NA in the control group and the SZ group. Table presents the data of the Kolmogorov-Smirnov statistics.  Table 2 (line 6) presents the descriptive statistics and comparison of two samples with *U* test. (c) The dependence of the cfDNA concentrations in blood plasma of the individuals on index NA. Green: control group; red: SZ group.

Figure 1(a) shows in the form of cumulative distributions the blood plasma cfDNA concentrations of healthy volunteers and schizophrenic patients. The SZ group distribution shifted towards large values of the cfDNA concentrations and differed from the HC group distribution (*p* < 0.001, Kolmogorov-Smirnov test).  Table 2 presents the descriptive statistics and comparison of two samples using the Mann-Whitney *U* test. The SZ group index cfDNA is higher than that in the HC group (*p* < 10^−4^). The median cfDNA values are approximately 30% higher in SZ patients than in the HC group. Therefore, the blood plasma of SZ patients contains more cfDNA than blood plasma of mentally healthy people.

Figure 1(b) shows endonuclease activity of blood plasma for two groups in the form of cumulative distributions; Table 2 presents the data of descriptive statistics. The distribution for the SZ group is shifted towards much larger values of index NA and significantly differs from the distribution for the HC group (*p* < 10^−27^). Approximately 70% of the SZ patients have index NA values greater than the HC group. Index NA for the SZ group is much higher than that for the HC group (*p* < 10^−28^; Table 2).

Figure 1(c) shows the dependence of the cfDNA concentration on the blood plasma endonuclease activity. We found a negative dependence of index cfDNA on index NA, more pronounced for the HC group.

### 3.2. cf-rDNA CN Is Increased in cfDNA of SZ Patients

**Figure 2:**
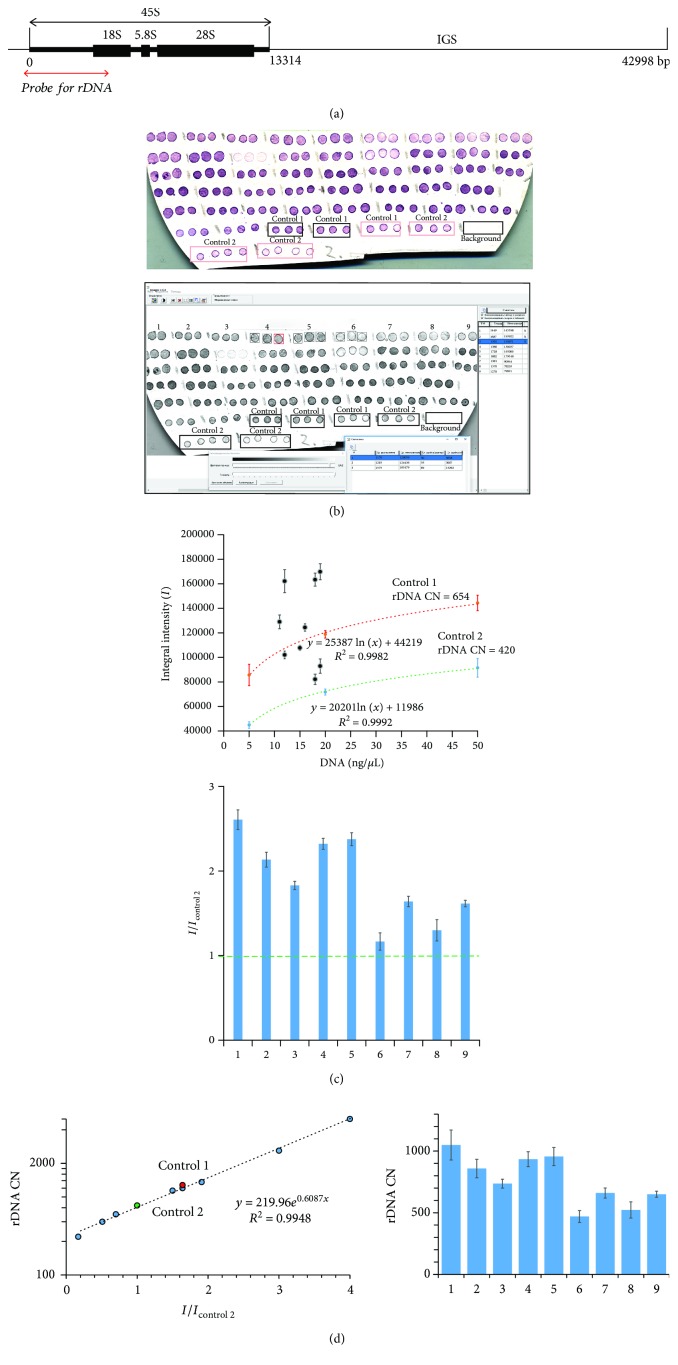
Determination of rDNA CN in human cfDNA using NQH. (a) Scheme of the human ribosomal repeat. Segment of rDNA analyzed with NQH is shown. (b) (1) Photo of the membrane fragments with visualized rDNA. Three spots are applied for each DNA sample. The calibration DNA samples containing 640 (control 1) or 420 (control 2) copies of rDNA were put onto these spots of the filter. (2) The filter was scanned and the average integral intensity *I* (±SD) of the spots was determined with software application “Imager 6.0.” (c) Dependence of *I* on the total cfDNA concentration in the sample plotted for the standard samples (dashed lines) and for the tested DNA samples 1-9 (as an example). (2) The ratio *I*_*i*_/*I*_control 2_ was calculated. (d) Calibration dependence of rDNA CN on the *I*_*i*_/*I*_control 2_ ratio plotted for DNA calibration samples with known rDNA CN. (2) The rDNA CN in the cfDNA was calculated.

The scheme of the rDNA copy number variation (CNV) analysis by NQH method is shown in Figure 2 and in Supplement.

**Figure 3:**
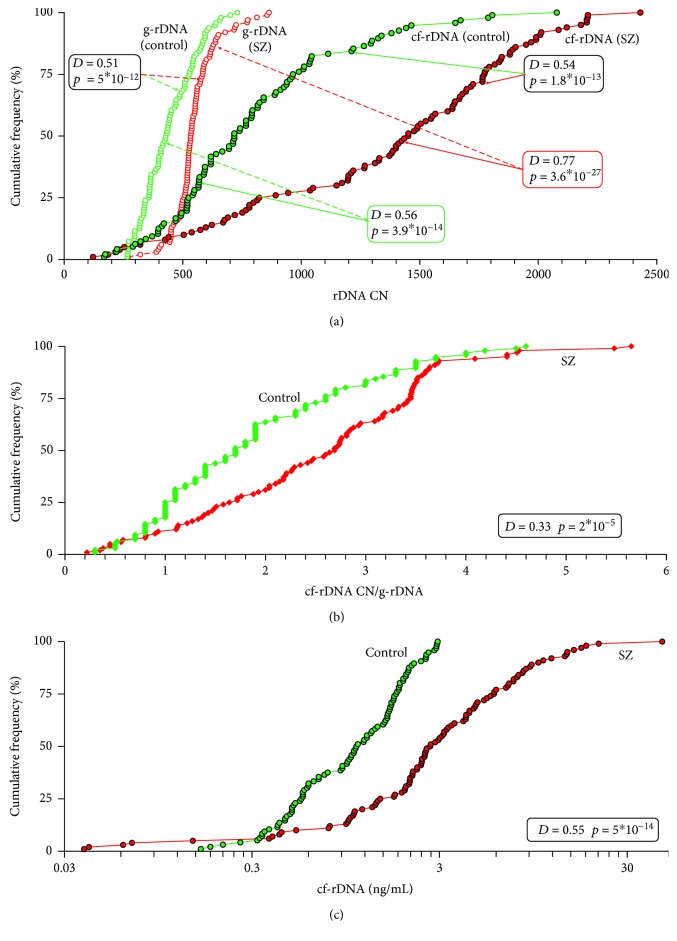
In the SZ male patients, the content of rDNA in the cfDNA and blood plasma increases. (a) Cumulative curves of the g-rDNA CN and cf-rDNA CN in the control group (*N* = 96) and the SZ group (*N* = 100). Tables on the graph presents the data of the Kolmogorov-Smirnov statistics for pairwise comparison of the two groups (compared groups are indicated by the arrows).  Table 2 (lines 3 and 4) presents the descriptive statistics and comparison of two samples using the *U* test. (b) Cumulative curves of the ratio *R* = cf-rDNA CN/g-rDNA CN in the control group and the SZ group. Table presents the data of the Kolmogorov-Smirnov statistics.  Table 2 (line 5) presents the descriptive statistics and comparison of two samples with *U* test. (c) Cumulative curves of the cf-rDNA concentration in the control group and the SZ group. Table presents the data of the Kolmogorov-Smirnov statistics.  Table 2 (line 2) presents the descriptive statistics and comparison of two samples with *U* test.

Figure 3(a) presents the content of rDNA in the DNA of white blood cells (g-DNA CN) and in the cfDNA of blood plasma (cf-rDNA CN) for the two groups in the form of cumulative distributions; Table 2 shows the descriptive statistics.

The distribution of g-DNA CN for the SZ group is shifted towards larger values and significantly differs from the distribution for the HC group (Figure 3(a)). Index g-DNA CN for the SZ group is much higher than that for the C group (*p* < 10^−9^) (Table 2). Thus, we confirmed previously obtained results [4]. Genomes of SZ patients contain an increased number of rDNA copies.

The distributions of cf-rDNA CN for the HC and SZ groups are shifted towards larger values and differ significantly from the distributions for g-rDNA CN in the corresponding groups (*p* < 10^−26^ and *p* < 10^−12^, respectively; Figure 3(a)). Thus, in both groups, we observed an increase in the rDNA content in the composition of cfDNA compared with the content of rDNA in the genomes of blood leukocytes.

The distribution of cf-rDNA CN for the SZ group is also shifted towards larger values and significantly differs from the distribution for the HC group (*p* < 10^−12^; Figure 3(a)). Index cf-rDNA CN for the SZ group is higher than that for the C group (*p* < 10^−11^; Table 2). Thus, the cfDNA of SZ patients contains more copies of rDNA than the cfDNA of healthy controls.

Figure 3(b) shows the enrichment coefficients for cfDNA with the rDNA fragments in the form of the ratio *R* = cf-rDNA CN/g-rDNA CN. In the SZ group, *R* values are higher than those in the HC group (Table 2; *p* < 10^−4^).

### 3.3. cf-rDNA Concentration Increases in Blood Plasma of SZ Patients

Figure 3(c) shows experimental values of plasma rDNA concentration for two groups in the form of cumulative distributions; Table 2 presents the data of descriptive statistics. The distribution of cf-rDNA for the SZ group is shifted towards larger values and significantly differs from the distribution for the HC group (*p* < 10^−13^). Index cf-rDNA for the SZ group is much higher than that for the C group (*p* < 10^−12^; Table 2).

### 3.4. Comparative Analysis of NA, cf-rDNA CN, cf-rDNA, and cfDNA

**Figure 4:**
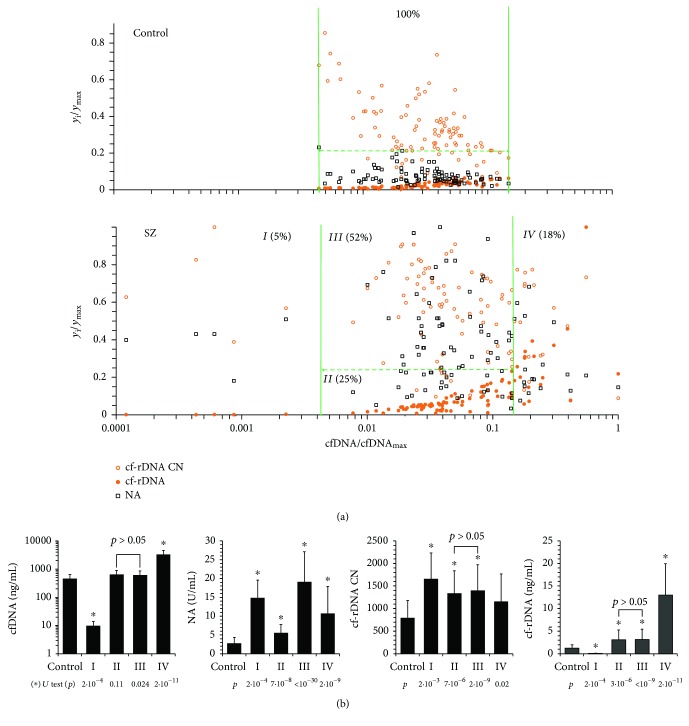
The characteristics of the cfDNA in patients with schizophrenia vary significantly as compared with the control. (a) Dependence of the relative cf-rDNA CN, cf-rDNA concentration, and NA values on the relative cfDNA concentration for the control and SZ groups. The relative values are obtained by means of dividing the respective value by the maximum value in each index group. The legend of the parameters is given in (a) in the box. The vertical and horizontal lines separate the values of relative cfDNA and NA parameters for the control group. (b) Comparison of cfDNA and cf-rDNA concentrations and index NA and cf-rDNA CN in SZ subgroups I-IV and the control group. *U* test data are given.

Figure 4(a) shows the dependence of the NA, cf-rDNA CN, and cf-rDNA on the cfDNA concentration for the HC group (*N* = 96) and SZ group (*N* = 100). All indicators were normalized in accordance with maximum values in two groups; therefore, their values ranged from 0 to 1. The cumulative data analysis allows distinguishing four subgroups (I-IV) in the SZ group.

#### 3.4.1. Subgroup SZ (I) (5% of the Total Sample)

Blood plasma of some patients contains a very low amount of cfDNA and, as a result, low amounts of rDNA. Possibly, the cells of these patients develop a response aimed at reducing the cell death, which we found earlier in some schizophrenia patients [12]. Apparently, the response does not develop immediately, as evidenced by the high values of NA and high (compared with the control) values of cf-rDNA CN.

#### 3.4.2. Subgroup SZ (II) (27%)

cfDNA concentrations and endonuclease activity vary within normal limits (HC group). cfDNA concentrations do not differ from the concentrations in the HC group (*p* > 0.1); however, endonuclease activity is significantly higher than that in the HC group, despite the same range of variation of the index NA in the control group and in the SZ group (Figure 4(b)). The number of cf-rDNA copies was increased (*p* < 10^−5^) in this subgroup in comparison with the control, and the concentration of rDNA was increased in blood plasma (*p* < 10^−5^). Thus, in SZ patients, even with the same total plasma concentrations of cfDNA, both rDNA content per unit of cfDNA and rDNA concentration change in blood plasma.

#### 3.4.3. Subgroup SZ (III) (52%)

cfDNA concentrations vary within the normal values (HC group) and do not differ from these parameters. Endonuclease activity is significantly higher than the upper values in the HC group. A significant increase in NA in this subgroup does not lead to a significant change in cf-rDNA CN and cf-rDNA concentration compared to the subgroup SZ (II) (Figure 4(b)).

#### 3.4.4. Subgroup SZ (IV) (18%)

This subgroup of patients is characterized by very high cfDNA concentrations, exceeding the upper limit of normal values. The NA values are higher than those in the HC group, do not differ from the values in the subgroup SZ (II), and are significantly lower than those in subgroups SZ (I) and SZ (III). Index cfDNA CN is lower than that in other subgroups and does not differ from that of the HC group. High cf-rDNA concentrations appear to be due to high concentrations of total cfDNA.

## 4. Discussion

In this study, we defined the characteristics of cfDNA in patients with schizophrenia and matched healthy controls. cfDNA levels were higher in schizophrenia patients than in healthy controls. This result is fully consistent with the result recently obtained by other authors [11] using fluorescence correlation spectroscopy and our own data obtained earlier with other samples of healthy people and patients with SZ [12].

An increase of the cfDNA concentration in the circulation is leading to a significant increase in endonuclease activity in the blood. We measured the endonuclease activity of blood plasma in the SZ group and found a 4-fold increase in NA in patients with schizophrenia compared to the control. This result indicates the increase in the level of cell death in the body of patients. Increased nuclease activity was found in a number of other diseases (acute myocardial infarction [25], pregnancy pathologies [28], and arterial hypertension [32]). In addition, a significant increase in NA was found in men, who contacted occupationally with the sources of external gamma/neutron radiation or internal *β*-radiation of tritium for a long time [27].

In patients with SZ together with an increase in the cfDNA concentration, cfDNA composition changes significantly. It is known that cfDNA may be enriched with GC pairs [19, 20], indicating the accumulation in the cfDNA composition of GC-rich genome fragments. Ribosomal repeat is a good marker of changes in GC composition of cfDNA because it contains a high number of GC pairs and is represented in the genome in hundreds of copies. We detected the increased content of rDNA in cfDNA of patients with SZ in comparison with controls. This fact is explained, firstly, by the high content of rDNA in the genomes of patients with SZ [4] and, secondly, by the increased resistance of rDNA to fragmentation in the presence of endonucleases [26]. An increase of the rDNA in the cfDNA and an increase in the total cfDNA concentration lead to the almost four times increase of rDNA concentration in the blood plasma of patients with SZ compared with the control. Previously, the same effect was observed in patients with acute myocardial infarction, coronary heart disease [25], and rheumatoid arthritis [16]. A significant increase in the cf-rDNA concentration and cf-rDNA CN was found in men for a long time working with sources of ionizing radiation [26].

**Figure 5:**
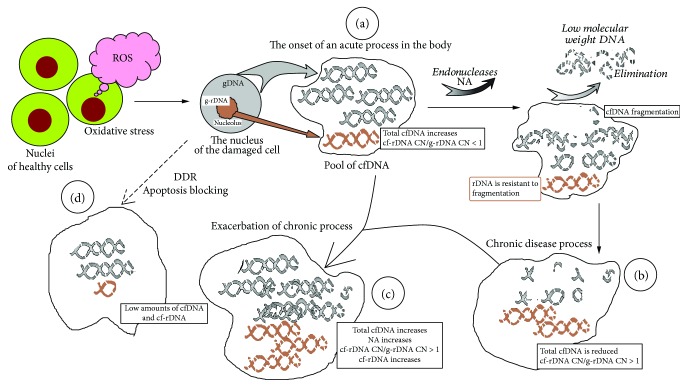
The scheme illustrates the change in cfDNA characteristics during acute and chronic oxidative stress. (a) The onset of an acute process in the body. Oxidative stress induces cell death. DNA of the dead cells appears in the circulation. Ribosomal DNA is localized in the closed structure of the nucleolus and remains highly molecular. Therefore, the content of the rDNA in the composition of cfDNA is less than in the genome. (b) In response to an increase in the concentration of cfDNA, the endonuclease activity of the blood increases. AT-rich DNA fragments are fragmented to low-molecular weight fragments and are eliminated from the bloodstream. Total cfDNA is reduced, but rDNA content is elevated. (c) If the chronic process is exacerbated, then additional cfDNA fragments appear in the circulation. Ribosomal DNA content is elevated. (d) Sometimes the cells develop the DNA damage response. Since the cells do not die, the pool of cfDNA is not replenished.

In general, the changes in the blood plasma of SZ patients were very similar to changes in the blood plasma of men exposed to low doses of radiation for a long time (gamma-neutron and beta-tritium radiation [26, 27]). This suggests a general pattern of changes in the characteristics of cfDNA under acute and/or chronic oxidative stress, regardless of endogenous or exogenous causes of this stress.  Figure 5 reflects our understanding of the processes leading to the changes in the characteristics of cfDNA in SZ patients.

### 4.1. State A: A Sharp Increase in ROS Levels in the Body

Schizophrenia is associated with elevated levels of ROS and DNA damage [7]. The reasons for the sharp increase in the level of ROS in the SZ patient are not yet clear. Emotional stress, environmental exposure, or biochemical abnormalities in the body's cell functioning may lead to an increase in ROS levels. In response to the action of ROS and DNA damage, the cells of the body die. The DNA of the dead cells is fragmented by cellular endonucleases and can then diffuse from the dead cells into the intercellular medium. Therefore, the amount of total cfDNA in the circulation increases significantly. The GC-rich rDNA is localized in the nucleolus. This genomic sequence is relatively slower cleaved into low-molecular fragments. The nucleolus is located in the central areas of the nucleus as a separate structure. These factors reduce the rate of the rDNA diffusion from the nucleolus into the medium. The content of rDNA in cfDNA is less than that in the gDNA of the cells (*R* < 1, state A in the diagram). State A, apparently, characterizes subgroup SZ IV (Figure 4): high cfDNA levels, relatively low NA, and low cf-rDNA CN values.

### 4.2. States B and C

Upon the DNA release into the extracellular medium, the cfDNA elimination system is activated. The endonucleases decompose the cfDNA to low-molecular fragments, which are excreted via the renal filtration later. The cf-rDNA is more resistant to the fragmentation and circulates in the form of high-molecular structures. As a result, the cf-rDNA fragments are accumulated in the blood plasma (*R* > 1) along with the decrease of the total cfDNA concentration. This condition is typical for subgroup SZ (III) and partly for SZ (II): the concentration of cfDNA is in the normal range with significantly increased NA and increased cf-rDNA CN and index cf-rDNA. Normal cfDNA concentrations together with high NA values indicate an active process of cfDNA release into the bloodstream (high level of cell death), which is balanced by the cfDNA elimination due to the high NA. Very high NA values and relatively high cfDNA values are indicated on state C with exacerbation of the chronic process.

### 4.3. State D

The low levels of cfDNA indicate a low level of cell death and the absence of large amounts of cfDNA in the bloodstream at the time of the study (subgroup SZ (I)). In our previous studies, it was shown that the low level of cfDNA in some patients correlates with a high level of DNA oxidation marker of cell nuclei. Cells with severely damaged DNA do not die that reflects a block of apoptosis [12] and DDR (DNA damage response).

## 5. Conclusion

Despite the abnormally high endonuclease activity in the blood plasma of SZ patients, the circulating cfDNA concentration is increased. Fragments of cf-rDNA accumulate in the blood plasma of SZ patients. Thus, analyzing the indices reflecting the properties of plasma cfDNA (cfDNA, cf-rDNA, cf-rDNA CN, and *R*) and endonuclease activity of blood plasma, it is possible to estimate the degree and duration of the pathological process in SZ patients. Potentially, SZ patients' cfDNA should be a strong stimulating factor for the TLR9-MyD88-NF-*κ*B signaling pathway. We plan to confirm this assumption in our future work.

## Figures and Tables

**Table 1:** Demographic and clinical measures in the male SZ patients and male HC.

#	Index		Control (*N* = 96)	SZ (*N* = 100)	*p*
1	Age	Mean	40.9 ± 15.1	37.4 ± 10.2	>0.05
		Median	39	35	
		Range	17-63	18-72	

2	Weight (kg)	Mean	70.4 ± 12.5	74.4 ± 13.9	>0.05
		Median	72	75	
		Range	65-131	50-162	

3	BMI (kg/m^2^)	Mean	26.2 ± 5.2	24.2 ± 4.3	>0.05
		Median	23.9	23.4	
		Range	17.1-38.2	17.9-54.8	

4	PANSS				
	Positive symptoms	Mean		25.7 ± 7.1	
		Median		25	
		Range		10-44	
	Negative symptoms	Mean		28.4 ± 9.3	
		Median		28	
		Range		0-49	
	General psychopathological symptoms	Mean		51.1 ± 13.2	
		Median		48	
		Range		24-97	

5	Smoking	Frequency			
	Never smoked	%	59	75	
	Stopped smoking less than 2 years ago	%	11	3	
	Smokes less than 20 cigarettes a day	%	18	12	
	Smokes more than 20 cigarettes a day	%	12	10	

**Table 2:** Some descriptive statistics for the parameters defined in the work.

#	Index		Control (*N* = 96)	SZ (*N* = 100)	*U* test *Z*; *p*
1	cfDNA (ng/mL)	Mean	461 ± 335	1069 ± 1723	3.78; 1.5*e*-05
		Coef.Var.	0.72	1.47	
		Median	409	539	
		Range	51-1698	1-11550	

2	cf-rDNA (ng/mL)	Mean	1.31 ± 0.81	4.83 ± 5.89	7.18; 6.6*e*-13
		Coef.Var.	0.61	1.22	
		Median	1.23	2.98	
		Range	0.16-3.79	0.04-46.14	

3	g-rDNA CN	Mean	441 ± 112	549 ± 96	6.36; 1.9*e*-10
		Coef.Var.	0.25	0.17	
		Median	430	534	
		Range	266-731	270-864	

4	cf-rDNA CN	Mean	796 ± 384	1356 ± 563	6.81; 9.5*e*-12
		Coef.Var.	0.48	0.42	
		Median	717	1452	
		Range	168-2078	121-2431	

5	*R*	Mean	1.91 ± 1.04	2.53 ± 1.14	3.99; 6.5*e*-05
		Coef.Var.	0.55	0.45	
		Median	1.70	2.69	
		Range	0.3-4.6	0.22-5.65	

6	NA (U/mL)	Mean	2.80 ± 1.57	14.00 ± 8.83	10.94; <1*e*-28
		Coef.Var.	0.56	0.63	
		Median	2.3	12.4	
		Range	0.6-8.8	1.3-38.1	

## Data Availability

The data used to support the findings of this study are included within the article.

## Supplementary Materials

Supplementary Materials Supplementary materials provide the protocols for nonradioactive quantitative hybridization (NQH). Materials also provide the calibration curve for the dependence of the signal intensity on the number of rDNA copies and DNA standard curve for cf-DNA concentration.
