# Supplementary material for: Accumulation of Circulating Cell-Free CpG-Enriched Ribosomal DNA Fragments on the Background of High Endonuclease Activity of Blood Plasma in Schizophrenic Patients
Source: Int J Genomics. 2019 Aug 5;2019:8390585. doi: 10.1155/2019/8390585 (PMC6701307; doi:10.1155/2019/8390585)
Supplement: Supplementary Materials — provide the protocols for nonradioactive quantitative hybridization (NQH). Materials also provide the calibration curve for the dependence of the signal intensity on the number of rDNA copies and DNA standard curve for cf-DNA concentration. [file 8390585.f1.pdf]

SUPPLEMENT

Nonradioactive quantitative hybridization (NQH)

For the detection of the human ribosomal repeat, the probe p(ETS-18S) (the fragment of rDNA 5.8-kb long, from −515 till 5321 relative to the transcription initiation point, HSU 13369, GeneBank) was used. It was cloned into the vector pBR322. The rDNA-probe was biotinylated using the nick translation kit (Biotin NT Labeling Kit, Jena Bioscience GmbH).

The membrane (ExtraC) was wetted with the solution of 10xSSC and dried. One (1) µl of 1M sodium hydroxide was added to 10 µl of the DNA solution in the TE-buffer at 0°C. The solution was mixed and incubated for 10 minutes at 0°C. Then, the DNA solution was neutralized with 11 µl of 20xSSC (pH = 5.0). The denatured DNA samples were applied to a filter in the amount of 1.5 - 2 µl per a dot. With respect to each sample, from 3 to 6 parallel dots were applied. Besides, standard genomic DNA samples were applied onto the same filter in order to define the calibration dependence of the signal on the number of the repeats in the sample. The DNA concentration in the standard calibration sample ranged within 0.5 - 50 ng/µl.

The filter was heated at 80°C in vacuum for 2 hours. In order to perform the hybridization, a special equipment was used, i.e. the hybridization furnace, which can vary the temperature and has a swaying table for mixing the solution above the membrane. The membrane was fixed in the solution (Denhard 10x solution; 0.05 M phosphate buffer, pH 7.0; 0.5 M sodium chloride; 50% formamide; 100 µg/µl tRNA E.coli) for 5 hours at 42°C. Then, the denaturated DNA probe in the concentration of 20 ng/µl was added. The hybridization was conducted for 16 hours at 45°C. The filter was washed with the solution of 2xSSC, 0.1 % SDS (2x15 min, 25 °C), 0.01 × SSC, 0.1 %SDS (20 min, 65°C) and 2xSSC (10 min, 25°C). After the hybridization the membrane was fixed (30 min; 37ºC) with a special solution (0.1% fat-free milk; 0.1% gelatin; tris-HCL buffer, pH 7.5; 0.1 M sodium chloride). Then, for 20 minutes (25°C) it was treated with the alkaline-phosphatase-conjugated streptavidin (1 µg/µl, Sigma) in a solution (0.1 M tris-HCL buffer; pH 7.5; 0.1 M sodium chloride; 0.005 M magnesium chloride). Then the filter was washed (3x10 min) with the following solution: (tris-HCL buffer pH 7.5; 0.1 M sodium chloride; 0.005 M magnesium chloride). After that, the filter was placed into the solution of substrates for alkaline phosphatase (tris-HCL buffer, pH 9.5; 0.1 M sodium chloride; 0.005 M magnesium chloride; 4.4 µg/µl NBT and 3.3 µg/µl BCIP). The reaction was performed in the darkness at 25°C, while visually

controlling the appearance of colored violet spots. Upon the end of the reaction, the filter was washed with water and dried in the darkness.

The dried filter was scanned. In order to perform the quantitative analysis of the hybridization result, it was used the software application “Imager 6.0” (MGNC RAMS). The software defines the spot location, the near-by background signal, as well as the surface area and integral intensity of the spot. The signals for a single sample are averaged; the average value and standard error are defined. The rDNA CN in a particular sample is calculated using the calibration curve equation. All the rDNA CN are calculated for the entire transcribed region of the ribosome repeat with the length of 13334 b.p.

Standard calibration curves.

Six standard samples of the genomic DNA (10 - 50 ng/mL) with a known content of the rDNA were applied to the same filter, in order to plot a calibration curve for the dependence of the signal intensity on the number of rDNA copies in a particular sample. The rDNA content the in standard calibration samples was detected earlier [Veiko et al., 2003]. To obtain the calibration samples with the higher content of rDNA than in the human genome, we added several picograms of model rDNA to samples of human DNA in the amount of several picograms per ng of DNA. Lambda phage DNA (10 - 50 ng/mL) was also applied to the same filter in order to control the nonspecific signal.

The dependence of the number of rDNA copies on the relative hybridization signal is well reproduced in independent experiments. Therefore, for each specific experiment, two calibration samples can be used instead of eight.

DNA standard curve for cf-DNA concentration.

The DNA concentration in the calibration sample was determined by UV spectrometry. DNA samples of varying concentrations were prepared by dilution.

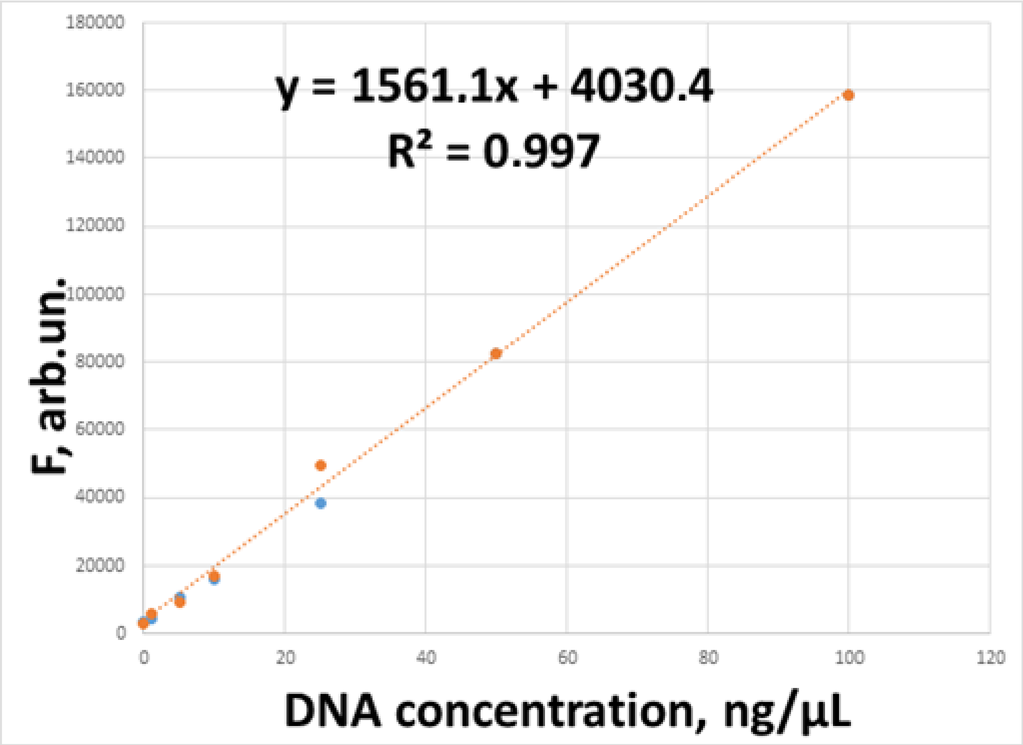
y = 1561.1x + 4030.4
R² = 0.997
F, arb.un.
DNA concentration, ng/µL

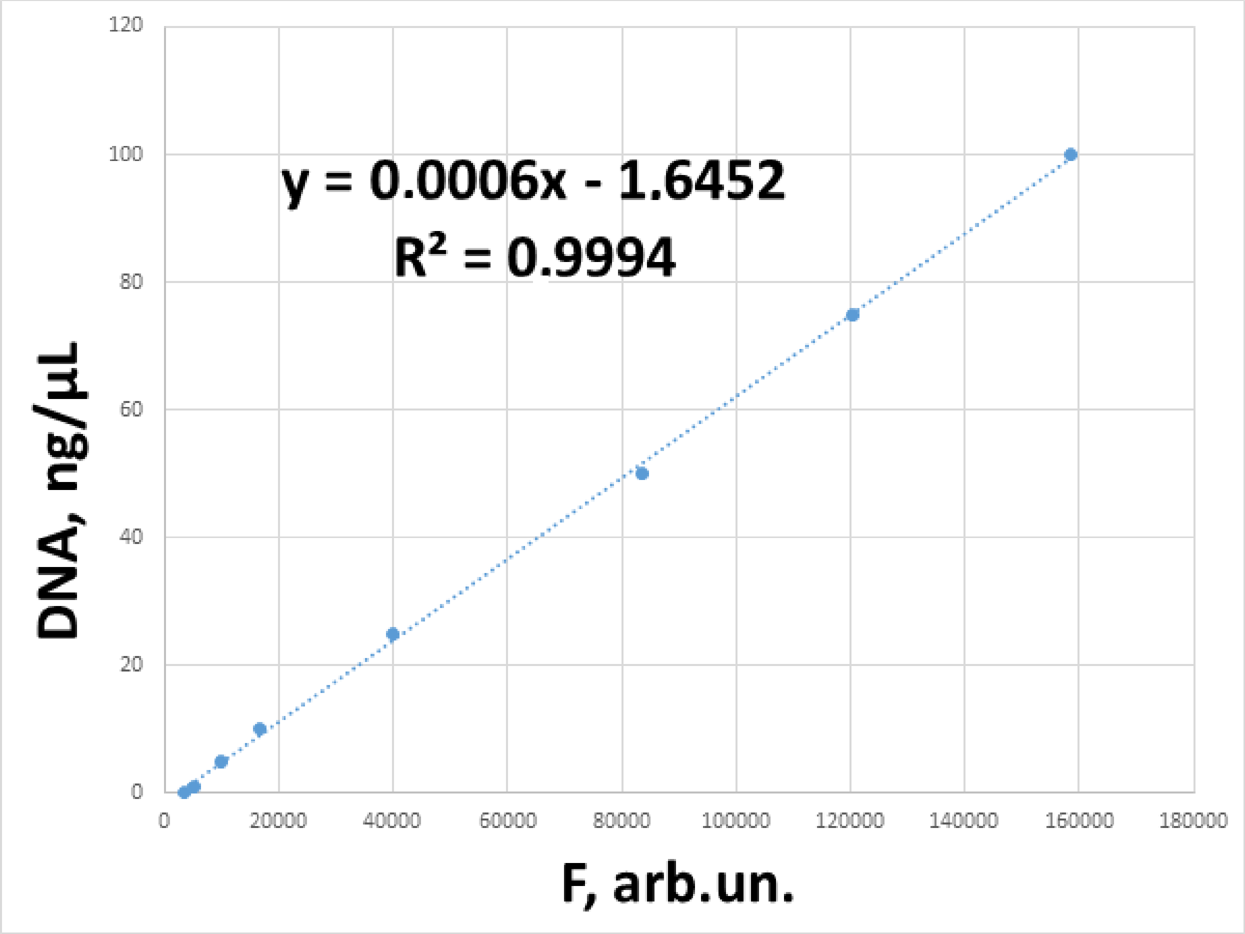
y = 0.0006x - 1.6452
R² = 0.9994
DNA, ng/µL
F, arb.un.
